# Effects of dietary *Antrodia cinnamomea* fermented product supplementation on antioxidation, anti-inflammation, and lipid metabolism in broiler chickens

**DOI:** 10.5713/ajas.19.0392

**Published:** 2019-08-26

**Authors:** M. T. Lee, W. C. Lin, L. J. Lin, S. Y. Wang, S. C. Chang, T. T. Lee

**Affiliations:** 1 Department of Animal Science, National Chung Hsing University, Taichung, 402, Taiwan; 2 School of Chinese Medicine, College of Chinese Medicine, China Medical University, Taichung 40402, Taiwan; 3 Department of Forestry, National Chung Hsing University, Taichung, 402, Taiwan; 4 Kaohsiung Animal Propagation Station, Livestock Research Institute, Council of Agriculture, 912, Taiwan; 5 The iEGG and Animal Biotechnology Center, National Chung Hsing University, Taichung, 402, Taiwan

**Keywords:** *Antrodia cinnamomea*, Broiler Chickens, Antioxidant, Microflora, Fatty Acids

## Abstract

**Objective:**

This study was investigated the effects of dietary supplementation of *Antrodia cinnamomea* fermented product on modulation of antioxidation, anti-inflammation, and lipid metabolism in broilers.

**Methods:**

Functional compounds and *in vitro* antioxidant capacity were detected in wheat bran (WB) solid-state fermented by *Antrodia cinnamomea* for 16 days (FAC). In animal experiment, 400 d-old broiler chickens were allotted into 5 groups fed control diet, and control diet replaced with 5% WB, 10% WB, 5% FAC, and 10% FAC respectively. Growth performance, intestinal microflora, serum antioxidant enzymes and fatty acid profiles in pectoral superficial muscle were measured.

**Results:**

Pretreatment with hot water extracted fermented product significantly reduced chicken peripheral blood mononuclear cells death induced by lipopolysaccharide and 2,2′-Azobis(2-amidinopropane) dihydrochloride. Birds received 5% and 10% FAC had higher weight gain than WB groups. Cecal coliform and lactic acid bacteria were diminished and increased respectively while diet replaced with FAC. For FAC supplemented groups, superoxide dismutase (SOD) activity increased at 35 days only, with catalase elevated at 21 and 35 day. Regarding serum lipid parameters, 10% FAC replacement significantly reduced triglyceride and low-density lipoprotein level in chickens. For fatty acid composition in pectoral superficial muscle of 35-d-old chickens, 5% and 10% FAC inclusion had birds with significantly lower saturated fatty acids as compared with 10% WB group. Birds on the 5% FAC diet had a higher degree of unsaturation, followed by 10% FAC, control, 5% WB, and 10% WB.

**Conclusion:**

In conclusion, desirable intestinal microflora in chickens obtaining FAC may be attributed to the functional metabolites detected in final fermented product. Moreover, antioxidant effects observed in FAC were plausibly exerted in terms of improved antioxidant enzymes activities, increased unsaturated degree of fatty acids in chicken muscle and better weight gain in FAC inclusion groups, indicating that FAC possesses promising favorable mechanisms worthy to be developed.

## INTRODUCTION

Poultry industry, especially meat, has occupied a leading role among agricultural industries; for it not only requires merely short rearing period, but provides high quality protein granting balanced amount of fatty acids [1]. However, there are two major factors impeding the development of poultry industry. Firstly, the availability of sufficient supplies of feed ingredients for the production of feeds is one of the major constraints [1], owing to the paucity of main raw materials resulting from intense grain crops requirement of biomass energy [2]. Agricultural byproducts, such as wheat bran (WB), thereby become promising candidates for the compensation. The WB is an important by-product of the cereal industry produced in vast quantities worldwide. Global demand for sustainable resources means that there is an increasing socioeconomic pressure to ensure complete utilization of such feedstock [3]. Secondly, oxidative stress has been evidenced to be strongly responsible for the impaired productive and reproductive performance and the consequent economic losses in poultry industry [4]. Moreover, literatures demonstrated that these stresses are able to accelerate the deterioration of lipid peroxidation and inflammatory responses that could be attributed to free radical production [5], implying that oxidation, inflammation, along with lipid metabolism are intertwined in animal body.

*Antrodia cinnamomea* (AC, *Syn. Antrodia camphorata* and *Taiwanofungus camphorata*) is a precious and unique medical fungus indigenous to Taiwan; and it is one of the member of wood-rotting basidiomycete that relies on efficient lignocellulose degradation in order to obtain nutrition for growth [6]. Attention has been brought by AC due to its curative properties originated from the realm of ethnic medicine, and those beneficial effects are related to various biological functions, including antioxidant, immunomodulatory, anti-cancer and hepatoprotective actions [7]. It is estimated that about 80% to 85% of all medicinal mushrooms’ products are derived from the fruiting bodies [8], which have been either artificially cultured or collected from nature. However, due to the increased in market demand and limited supply of natural source, mycelial products, produced from solid state or submerged liquid fermentation, are therefore considered the wave of the future [7]. Chang [8] found that the extracts of solid-state cultured AC enhanced the anti-proliferative effects of co-administrated anti-tumor agents, which could be attributed to the antioxidant and antitumor properties of AC extract. Considering the pragmatic application in animal field, WB is recognized as a decent substrate. Low-cost as it possesses, WB, still, is regarded as undesirable materials in diet due to the non-starch polysaccharides (NSPs) in it tend to reduce their digestibility of monogastric animals. Chen et al [9] used various NSP rich cereal to induce gut inflammatory in broiler chickens in order to establish an effective way to mitigate related gut illness. Since AC possesses ability to utilize such lignocellulosic material, employing WB as substrate for AC to grow provides strategies to not only generate promising bioactive feed material, but also eliminate adverse effects of WB and elevate the value of such agricultural byproduct.

Several research has established the possible consequences under specific stress condition. Pervasively, heat stress [4], intended challenge chickens with common pathogens or stress hormone [10], and even overcrowding stress [11] tend to cause severe deprivation of antioxidant capacity, increased lipid peroxidation level, undesirable lipid metabolism, jeopardized antimicrobial immunity and even compromised growth and reproductive performance. Nonetheless, given that dealing with the symptom instead of the core problem is not a long term solution, and few research had conducted to delineate the effects of specific nutritional regimens on the stress response which may occur in practical farm, nutritional manipulation may be a decent approach to address and even prevent the central issue effectively.

Accordingly, the object of the present study was therefore to investigate the antioxidant, indirect immunomodulation, and lipid regulation effects of solid-state fermented WB with AC in broiler chickens under conventional rearing condition.

## MATERIALS AND METHODS

### Organism culture of *Antrodia cinnamomea*

One of the examined AC mycelia was kindly provided from Department of Forestry at National Chung Hsing University, Taichung, Taiwan (Dr. Sheng-Yang Wang lab). Culture was maintained in crystalprotective tube containing malt extract broth (MEB, glucose 2%, malt extract 2%, peptone 0.1%) with 30% glycerol, and stored at −80°C. To prepare the inoculum, the mycelium of AC was transferred to petri-dish containing malt extract agar (MEA, glucose 2%, malt extract 2%, peptone 1%, and agar 2%) medium at 28°C for 20 days.

### Inoculum preparation of *Antrodia cinnamomea*

The basal medium for shake flask culture was made up of MEB, the pH was initially adjusted to 5.0 before sterilizing by autoclaving (at 121°C±1°C for 30 min). AC was transferred to the medium by punching out agar cubes (about 1 cm×1 cm) from culture grown on MEA plates, and 20 pieces of it were used to inoculate in 100 mL of liquid media. The seed culture was grown in a 250 mL Erlenmeyer flask with an air-permeable sponge-like plugs at 28°C on a rotary shaker incubator at 140 rpm for 5 days.

### Solid-state fermentation

Solid-state culture of AC was performed in plant tissue culture glass bottle with silicone cap, kindly landed from the R&D Center of Taiwan Leader Biotechnology Corp. (Taichung, Taiwan), containing 50 g WB (Formosa oil seed processing Co. Ltd., Taichung, Taiwan) as substrates. Moisture content was adjusted to 45%, and then thoroughly mixed and sterilized at 121°C±1°C for 30 min in an autoclave. Until the glass bottles cooled, a 10% inoculum was conducted. The bottles were incubated at 28°C for 20 days, and the final products were procured after 16 days fermentation period. The sample cultured using strain from Wang’s lab named Wang’s *Antrodia cinnamomea* in the following content.

### *Antrodia cinnamomea* mycelial powder

Solid-state cultured *Antrodia cinnamomea* mycelia powder (ACP), which had been certificated as Health Food (A00190) by the Department of Health, Taiwan, was obtained from the R&D Center of Taiwan Leader Biotechnology Corp. (Taiwan).

### Preparation of fermented *Antrodia cinnamomea* extract

To prepare extracts of ACP and WAC, 5.0 g samples were weighed and incubated with distilled water in flasks, then and left to stand at 95°C for 1 h. The solution was then quantified to 100 mL. The extracts obtained were centrifuged at 3,000 rpm for 10 min and filtered through No. 1 filter paper and then stored at −20°C for subsequent analysis.

### Total phenolic content

Briefly, an aliquot of 50 μL submerged culture broth/extract was mixed with 0.5 mL Foline-Ciocalteau phenol reagent and 1 mL 7.5% sodium carbonate, and allowed to react for 30 min at room temperature (RT) comparative to a gallic acid standard. The absorbance was measured at 730 nm using an automated microplate reader (Sunrise, TECAN, Zürich, Switzerland). The results were expressed as gallic acid equivalence.

### Total polysaccharides

The water-soluble polysaccharides were precipitated by adding 4 volumes of 95% ethanol. The precipitated polysaccharides were collected via centrifugation at 3,000 rpm for 10 min, and subsequently dried at 60°C to remove residual ethanol. Total amount of polysaccharides in the culture broth or extract was determined by phenol-sulfuric acid assay, *as per* Dubois et al [12] 1 mL samples were pipetted, respectively, into dilution tubes. One mL 5% (v/v) phenol and 5 mL of 18 M sulfuric acid was then added and the mixture was left to stand for 10 min at RT. The tubes were then vortex thoroughly and left for 30 min at RT, and then immersed the tube into ice-cold water to cease the reaction. The absorbance was measured at a wavelength of 490 nm using a spectrophotometer (Ultrospec 2100 pro, mersham, Hong Kong). A blank test was prepared by substituting distilled water for the sugar solution, and the standard curve was prepared using glucose in order to calculate the relative amount of polysaccharide in samples.

### Crude triterpenoid content: Sample extraction

Four g samples were weighed into a 50 mL centrifuge tube and mixed with adequate amount of 95% ethanol for proper dilution, shaking at 200 rpm for 30 min, and further extracting by ultrasonication (DC 300, DLETA, Taipei, Taiwan). After centrifuge the tube at 5,000 rpm for 5 min, the supernatant was collected for the subsequent analysis.

### Crude triterpenoid analysis

Briefly, 100 μL sample was put into a dilution tube, and then place it into a water-bath for evaporating the ethanol. One hundred and fifty μL mixed 5% (w/v) vanillin-acetic solution and 500 μL perchloric acid were added, covered the tube with aluminum foil, mixed and incubated at 60°C for 20 min. The solution was then cooled and added 5 mL acetic acid. The absorbance was measured at 573 nm using a spectrophotometer. A blank test was prepared by substituting distilled water for the reagents apart from sample, and the standard curve was prepared using ursolic acid (CAS 77-52-1, Sigma, Darmstadt, Germany) in order to calculate the relative amount of triterpenoid in samples.

### Ferrous chelating capacity assay

In brief, 250 μL submerge culture broth/extract was mixed with 25 μL 2 mM ferrous chloride solution and 925 μL methanol. After 30 min at RT, 50 μL 5 mM ferrozine was added to initiate a reaction that lasted for 10 min at RT. The absorbance was then determined at 562 nm. The inhibiting percentage of ferrozine-Fe^2+^ complex formation was calculated as:

$$\text{Ferrous ion chelating }(\%)=\left(\frac{A_{0}-A_{1}}{A_{0}}\right)\times 100$$

where A_0_ and A_1_ represents the absorbance of the control and sample, respectively. In this experiment, ethylenediaminetetraacetic acid (EDTA) was used as a positive control.

### Scavenging effect on 1,1-diphenyl-2-picrylhydrazyl radicals

Two mL of each extract sample was mixed with 500 μL 1 mM 1,1-diphenyl-2-picrylhydrazyl (DPPH) ethanol solution. The mixture was shaken vigorously and left to stand for 30 min in the dark; the final absorbance was then measured at 517 nm against a blank. The values were converted into the percentage scavenging activity using the following formula:

$$\text{Scavenging activity }(\%)=\frac{(\text{Abs sample}-\text{Abs blank})\times 100}{\text{Abs control}}$$

Butylated hydroxytoluene (BHT) was used as a positive control in this assay.

### Chicken peripheral blood mononuclear cells isolation

Whole blood of chicken was collected via wing-vein using a hypodermic syringe and inserted into tubes containing EDTA. The blood was gently layered on to Ficoll-Paque Plus and centrifuged at 200×g for 10 min. Chicken peripheral blood mononuclear cells (cPBMCs) were collected from the gradient interface; the plasma suspension was combined and washed three times with phosphate-buffered saline and then centrifuged at 200×g for 10 min. After the suspension was removed, RPMI-1640 was used as the solvent for adjusting cell count to 10^8^ cell/mL, which were pipetted 2 mL cell suspension into 6 well plates and cultured at 37°C in 5% CO_2_ mixed with 95% air in an incubator for 2 h. After incubation, the cells were treated with hot water extracted fermented product (HFAC) (25, 50, 75, and 100 mg/mL) in the presence of 2,2′-Azobis (2-amidinopropane) dihydrochloride (AAPH) (10 mM) or lipopolysaccharide (LPS) (100 ng/mL) for 24 h. Pipetting the whole culture liquid into a sterilized tube, and centrifuged at 200×g for 10 min. One mL Trizol reagent was added and the mixture was stored at −80°C.

### Cell viability - colorimetric MTT assay

The cell viability assay was determined by 3-(4, 5-dimethylthiazol-2-yl)-2, 5-diphenyl tetrazolium bromide (MTT, Sigma-Aldrich, Germany). Briefly, cPBMCs were seeded in 96 well plates. After 24 h incubation, HFAC dilutions were added to each well and incubated for 48 h at 37°C in 5% CO_2_. Afterward, the supernatant was discarded, and then added 28 μL of MTT solution (2 mg/mL in phosphate buffered saline). The plates were incubated at 37°C for 2 h, and then dissolved the resulting formazan crystals with 130 μL dimethyl sulfoxide (DMSO). Finally, the optical density was measured at 517 nm wavelength.

### Experimental birds and housing

Four hundred 1-d-old male broiler chickens (Ross 308) were evenly divided by weight (approximately 41 to 41.5 g/bird) and then randomly allocated to one of the five treatments. Each treatment group had four replicates per pen, with 20 birds per pen (totaling 80 birds per treatment).

### Environmental factors

The temperature was maintained at 34°C±1°C until the birds reached 7 days of age; it was then gradually decreased to 26°C ±1°C until the birds reached 21 day of age. After this point, the broilers were maintained at RT (approximately 27°C). The experiment was conducted at the ranch of National Chung Hsing University, Taiwan, and the experimental protocol was approved by the Animal Care and Use Committee (IACUC No. 102–126).

### Dietary treatment and feeding schedule

The birds in the control group received corn-soybean meal basal diet; the 5% WB group was fed the basal diet with 5% replacement of WB; the 10% WB group was fed the basal diet with 10% replacement of WB; the 5% FAC group was fed the basal diet with 5% replacement of FAC; and the 10% FAC group was fed the basal diet with 5% replacement of FAC (Table 1).

### Feeding schedule

All birds received starter (1 to 21 days of age) and finisher (22 to 35 days of age) diets *ad libitum* and had free access to water. The proximate composition of the diets was analyzed according to the AOAC (2000). Crude protein, crude fat, ash and acid detergent fiber levels were determined using methods 990.03 (Kjeldahl N×6.25), 945.16, 967.05, and 973.187, respectively; the results showed no major deviations from the calculated values. During the entire experimental period (35 days), the diets were formulated to meet the requirements suggested by the Ross Broiler Management Manual [13] and the NRC [14].

### Performance and sample collection

Body weights were recorded at 1, 21, and 35 days of age. Body weight gain and feed conversion ratio (FCR) were calculated on the basis of the above data. At 21 and 35 d, eight birds (two birds per replicate) were randomly selected for sampling. Blood samples were collected via wing-vein puncture into a tube containing 1% EDTA. The samples were then centrifuged at 3,000×g for 10 min to obtain the serum, and the aliquots were transferred into microfuge tubes. Sera were kept on ice and protected from light to prevent any oxidation during sample collection. Samples were stored at −20°C until analysis. The birds were euthanized by electrical stunning for extermination, and then dissected to collect the breast muscle (pectoral superficial muscle) and then the abdominal cavities were opened for liver, ileum and ceca collection. The breast muscle samples were immersed in liquid nitrogen immediately after took out of abdomen, and then stored at −80°C until analysis; the ileal and cecal contents were collected for the study below.

### Determination of ileal and cecal microbial population

Lactic acid bacteria and coliform were cultured with De Man, Rogosa and Sharpe agar and Chromocult Coliform Agar medium, respectively. After aerobic and anaerobic incubation, respectively, at 37°C for 48 h, the microflora numbers were calculated. Bacterial populations were expressed as log10 colony forming units per gram of intestinal contents.

### Determination of serum antioxidant enzyme activities

Total superoxide dismutase (SOD) and catalase (CAT) activities were assayed using kits purchased from Cayman Chemical Co., Ltd. (Ann Arbor, MI, USA) Serum samples were measured in triplicate and at the appropriate dilutions to allow enzymatic activities to achieve the linear range of standard curves. Antioxidant enzyme activities were expressed as unit per milliliter of serum.

### Blood characteristics

At the end of the experiment, four broilers were randomly selected from each treatment (one birds per pen) for sampling. The blood samples were collected by jugular vein puncture from each bird, and then stored at −20°C until analysis. Samples for serum analysis were centrifuged at 3,000×g for 15 min to separate the serum. The concentration of total cholesterol (CHO), triglyceride (TG), low-density lipoprotein (LDL) cholesterol, and high-density lipoprotein (HDL) the serum samples were analyzed with an automatic biochemical analyzer (Hitochi, 7150 auto-analyzer, Hitachi, Tokyo, Japan).

### Fatty acids profile in pectoral muscle of chickens

The extract from chicken muscle was prepared from 10 g homogenized meat with chloroform/methanol (2:1 v/v). Fatty acid methyl esters (FAME) were prepared by following the procedure of Peisker [15]. The analysis of FAME was performed on a Shimadzu GC-2010 gas chromatograph (Shimadzu Co., Kyoto, Japan) equipped with a flame ionization detector. The separation of methyl esters of fatty acids was carried out on a capillary column (Alltech FAME, 30 m long, 0.32 id, 0.25 μm film thickness). Nitrogen was used as carrier gas (1.41 mL/min). The injection port temperature was 250°C and the detector temperature was 275°C. The column was operated at 150°C for 0.5 min, and then the temperature was increased to 180°C at 10°C/min and straight to 220°C at 3°C/min. Finally, temperature was increased to 250°C at 10°C/min. and held at 250°C for 10 min. Individual FAME peaks were identified by comparing their retention times with those of standards (FAME Mix 37, Supelco, Bellefonte, PA, USA). Results are calculated by established program by calcuating retention times and peak area percentages, and further expressed as percentage of total fatty acids.

### Statistical analysis

The data were analyzed by performing analysis of variances for completely randomized designs and executing the general linear model procedure implemented in SAS software.

## RESULTS

### Bioactive compounds in products solid-state fermented wheat bran by FAC

Table 2 shows three main crude metabolite content in FAC. The crude triterpenoid, total polyphenol, and crude polysaccharides content of FAC was 20.2±1.1 mg/g dry weight (DW); 16.7±0.7 mg/g DW; 123.3±3.1 mg/g DW, respectively.

### *In vitro* antioxidant capacity

**Figure 1:**
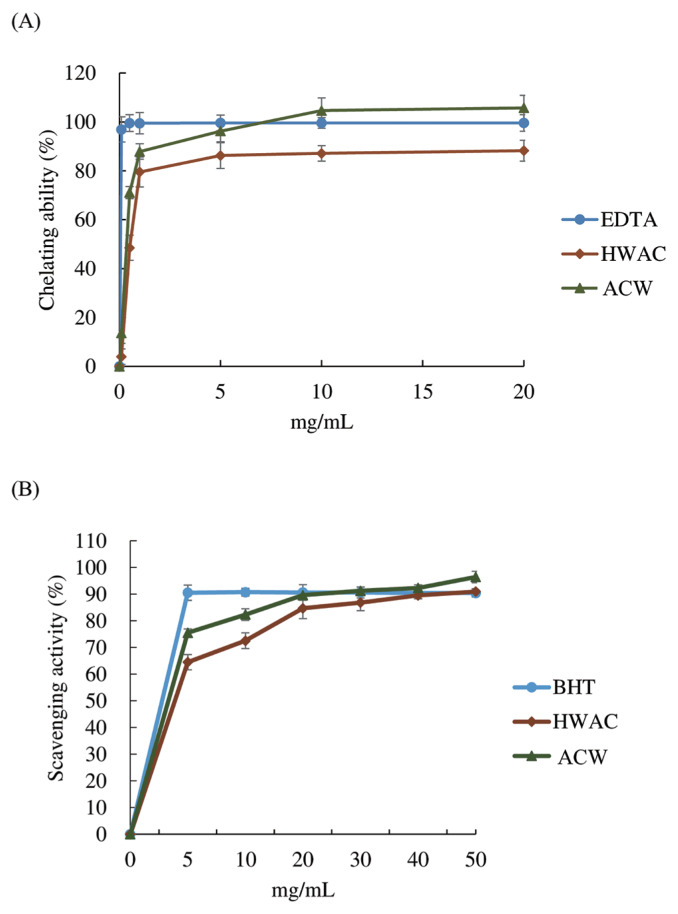
Antioxidant capacity includes (A) ferrous chelating and (B) DPPH scavenging capacity of hot water extracted product solid-state fermented by *Antrodia cinnamomea* for 16 days. Values are expressed as the mean±standard deviation of three samples (n = 3). HFAC, hot water extracted product solid-state fermented by *Antrodia cinnamomea* for 16 days; ACW, hot water extracted solid-state cultured *Antrodia cinnamomea* mycelia powder.

Figure 1A and 1B shows the ferrous chelating capacity and DPPH free radical scavenging ability of HFAC respectively. In these experiments, Water extracts of hot water extracted solid-state cultured *A. cinnamomea* mycelia powder (ACW) was also analyzed as a comparison. Despite lower chelating action in comparison with AC control (97.6%) was found in HFAC, it nearly saturated at 5 mg/mL with an equivalent 82.5% chelating effect, and exerted comparable effects even at higher concentration (Figure 1A). On the other hand (Figure 1B), within the analyzing spectrum, BHT peaked at 5 mg/mL without further increase with higher concentration. HFAC at 5 mg/mL exhibited 63.8% equivalent scavenging activity, along with 72.9% equivalent scavenging activity of ACW. However, the following logarithmic increment had HFAC possess commensurate scavenging activity with BHT at 40 mg/mL, and ACW showed 97.87% equivalent scavenging effect at 20 mg/mL.

### Effect of HFAC on cell viability of chicken peripheral blood mononuclear cells

**Figure 2:**
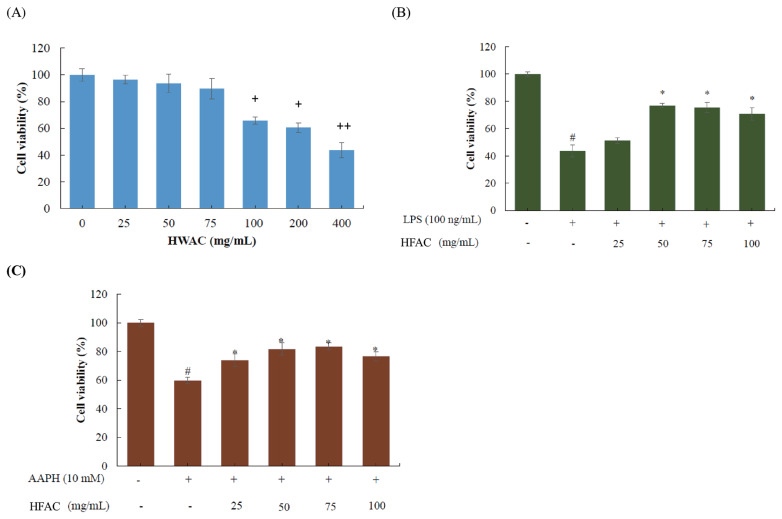
Evaluation of cytotoxicity and protective effects of hot extracted product solid-state fermented by *Antrodia cinnamomea* for 16 days (HFAC) toward (A) chicken blood mononuclear cells (cPBMCs) per se, (B) LPS-stimulated cPBMCs, and (C) AAPH-stimulated cPBMCs. Values are expressed as the mean±standard deviation of six samples (n = 6). HFAC, hot water extracted product solid-state fermented by *Antrodia cinnamomea* for 16 days; cPBMCs, chicken blood mononuclear cells; AAPH, 2,2'-Azobis(2-amidinopropane) dihydrochloride; LPS, lipopolysaccharide. ^+^p<0.05 was considered significant for other concentrations versus 0 mg/mL HWAC. ^++^p<0.05 was considered significant for other concentrations versus 0 mg/mL HWAC. ^#^p<0.05 was considered significant for control versus AAPH or LPS. * p<0.05 and ** p<0.01 were considered significant for sample versus AAPH or LPS.

Cytotoxic effects of HFAC on cPBMCs were examined using the MTT colorimetric assay in order to understand the potential negative or positive effects prior to the following *in vitro* studies (Figure 2). As shown in Figure 2A, there were no cytotoxic effect observed as cPBMCs treated with 25 to 75 mg/mL for 24 h. While at concentrations greater than 75 mg/mL, cell viability was suffered and decreased to only 43.68% as co-cultured with 400 mg/mL HFAC. Hence, concentrations of HFAC ranging from 25 to 100 mg/mL were used for further experiments.

Protective effects of HFAC were investigated and showed in Figure 2B and 2C. Decreased cell viability in LPS-induced cPBMCs (40.65%) was significantly inhibited by 50 to 100 mg/mL HFAC. Additionally, similar negative action exerted by AAPH (10 mM) was also ameliorated by HFAC in terms of improved cell viability as adding 25 to 100 mg/mL HFAC.

The above results indicate promising protective effects of HFAC against LPS- and AAPH-induced cell death.

### Growth performance

Table 3 shows the effect of dietary replacement with WB or FAC on growth performance of broiler chickens. In the starter phase (1 to 21 d), chickens received 10% FAC diet showed the greatest body weight and the most feed intake among all groups; by contrast, chickens in 10% WB had the lightest body weight and the lowest feed consumption. Moreover, birds in 5% FAC and 10% FAC group exhibited a comparable weight gain to those in the corresponding control group, which showed significantly higher weight gain than those in 5% and 10% WB groups. Collectively speaking, chickens had 10% FAC in diet performed better FCR in comparison with control group that had no difference with the results of 5% WB group; and 10% WB plus 5% FAC groups both showed similar FCR to others.

For the finisher phase (22 to 35 d), as comparing to other three groups, chicken diet contained WB in either replacement ratio group had birds suppressed body weight, and poor feed consumption as well; these ended up with lower weight gain in these groups. Despite showing lower feed consumption than control and 5% FAC group, 10% FAC still performed commensurate body weight and weight gain with 5% FAC, and showed no difference with control group.

Regarding the overall growth period (1 to 35 d), 5% and 10% FAC replacement in control diet exerted no adverse effects on growth performance in terms of comparable feed intake and body weight gain to those in control group. Nonetheless, chickens received diet with 5% and 10% WB replacement demonstrated poorer feed consumption and weight gain, even though no difference was found in FCR among each group.

### Selected microbial count in ileum and ceca

Effect of dietary replacement with WB and FAC on intestinal bacteria, lactic acid bacteria and coliform stand as representative, on broiler chickens were presented in Table 4. Ileal lactic acid bacteria count increased as diet containing 10% FAC in chickens aged 21, followed by 5% FAC, and control and 10% WB dietary treatment group. Besides, ileal lactic acid bacteria in 5% WB group showed only lower than 10% FAC group. On the other hand, cecal lactic acid bacteria count of chickens in 5% and 10% FAC group elevated significantly than the result in control group at both analyzed age (21 d- and 35 d-old), but 10% WB showed no difference with 5% FAC at 21 d. At 35 d, lower cecal lactic acid bacteria count was observed in 5% and 10% WB group.

Notwithstanding that no significant difference was ob served among each group in regard to ileal coliform count in both ages, either group containing WB had higher cecal coliform count at ant testing age. Supplementation of 5% FAC in diet had chicken the second most cecal coliform count at 21 and 35 d, but at the latter time, it presented similar count to 10% FAC and control groups.

### Serum antioxidant enzyme activities

**Figure 3:**
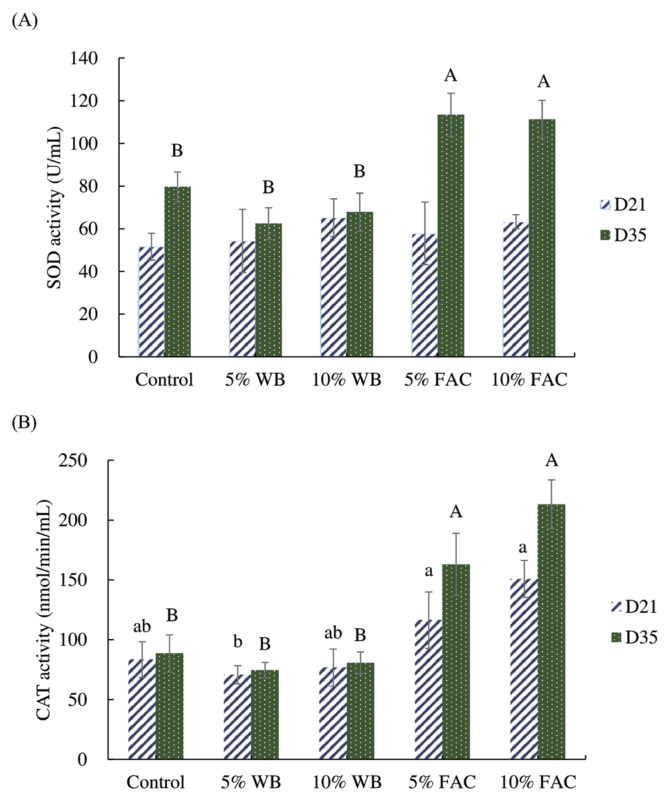
Effects of dietary replacement with product solid-state fermented wheat bran by *Antrodia cinnamomea* for 16 days (FAC) on (A) superoxide dismutase (SOD) and (B) catalase (CAT) in the serum in broilers aged 21 and 35. Values are expressed as the mean±standard deviation of eight samples (n = 8). WB, wheat bran; FAC, products solid-state fermented wheat bran by *Antrodia cinnamomea* for 16 days. ^a,b^ Means among groups in 21-day without the same letter are significantly different (p<0.05). ^A,B^ Means among groups in 35-day without the same letter are significantly different (p<0.05).

Effects of dietary replacement with WB and FAC on the serum antioxidant enzyme activities in chickens were shown in Figure 3. Among groups, no statistical difference was found in SOD activity in 21-d-old broiler chickens; but until 35 d, SOD activity in chickens fed 5% and 10% FAC containing diet was superior to three other groups. Similar boosting manner was also exerted in the CAT activity of chickens aged 35. Regarding the CAT activity in 21-d-old chickens, those received 5% and 10% FAC diet showed higher levels than that in 5% WB, and statistically allied activity was found in control and 10% WB groups, showing no difference with other groups.

### Serum lipid parameters

The effect of dietary replacement with WB or FAC on serum lipid parameters is shown in Table 5. In regard to broilers on 21 d old, chicken received 10% FAC diet exhibited the lowest TG level, followed by 5% FAC and 10% WB, and with 5% WB and control group showed highest content. LDL in 10% FAC group showed similar pattern to TG, except that 5% FAC had comparable low level of LDL with 10% FAC, along with 5% WB and 10% WB. On the contrary, highest HDL was rather found in birds consumed 5% FAC, 10% FAC, and control diet as well, suggesting that those had WB in diet showed relative low level of HDL.

Furthermore, for chickens at 35 d old, 10% FAC group showed the relative lowest level of CHO and LDL as comparing with, except for 5% FAC regarding LDL, others; while 5% WB group had the highest level of these two parameters in average. In TG, control diet group have chickens showed the highest level of it, followed by 10% WB, and 5% WB as well as 10% FAC; for those chickens had 5% FAC in diet rather had the lowest TG content. Finally, HDL index shared similar pattern with those observed at 21 d, only that the control group showed a comparable low level of HDL with that in chickens had WB in diet.

### Fatty acids composition of broiler breast meat

Table 6 demonstrated the effect of dietary replacement with WB or FAC on fatty acid composition in pectoral superficial muscle of 35-d-old broiler chickens. Even though there were only 4 fatty acids, that is palmitic acid (C16:0), stearic acid (18:0), oleic acid (18:1n9), and linoleic acid (18:2n6), exhibited mainly in the meat samples, significant difference was also found in other detected fatty acid index. For those 4 fatty acids, saturated form fatty acids, namely palmitic (C16:0) and stearic (C18:0) acid, were consistently lower in 5% and 10% FAC groups than others, especially 10% WB group. On the other hand, unsaturated oleic (C18:1n9) and linoleic acid (C18:2n6) were expressed the lowest amount in 10% WB group, with groups received FAC demonstrating the highest level by contrast. Additionally, palmitoleic (C16:1) and arachidonic (C20:4n6) showed pattern in line with each other that 5% and 10% FAC had chickens had the highest level of them; and others expressed relatively low concomitantly. Two forms of linolenic acid (C18:3n6, 3), despite no exact the same statistical results, were increased in birds fed FAC included diet in comparison with the corresponding control group; and 5% and 10% WB groups had no difference with each other in both index, and that they were both as low as the control group in α-linolenic acid (C18:3n3). Concerning the saturation degree among the five groups, it’s obvious that 5% FAC diet would have chickens much higher degree of unsaturation, followed by 10% FAC, control, 5% WB, and 10% WB.

## DISCUSSION

In the present study, three major metabolites of AC were detected to evaluate the promising function of our FACs. Beneficial bioactivities of AC have been studied by how a specific compound of AC acts and its further application to human healthcare regimen. Yang et al [16] employed four cereal grains as AC growing substrate, suggesting that secondary metabolites such as phenolic compounds increased up to 9.67 mg/g DW. maximum as comparing with the unfermented material, triterpenoids content was also evaluated in this study. This phenomenon indicate that substrate provides nutrients to AC for growth and metabolization, thus releasing active components afterward. Our study further assessed the potential antioxidant effects of the FAC. The chelating ability of and DPPH free radical scavenging abilities showed 80.3% and 94.6% of the antioxidant capacity of ACP respectively. Several studies applied organic solvents to demonstrate the antioxidant capacities of AC. For example, methanol extract of solid-state fermented AC product in research done by Yang et al [16] suggested comparable DPPH scavenging abilities to the butylated hydroxyanisole, an artificial antioxidant, at a relatively low extract concentration.

Macrophage proliferation is a way to assess the innate im mune augmentation effect of testing sample, and the outcome is directly correlated with the host defense capability against microbial pathogens. In the present study, decreased cell viability under LPS and AAPH challenge in cPBMCs was remarkably ameliorated by HFAC, with 50 mg/mL had the most effective improvement in average. LPS administration has been applied to set as an optimal model for study inflammation in cells [5]; while AAPH is an azo compound widely used to study the antioxidant effect of certain material since it tends to stimulate oxidative damage in cells [17]. In other words, these suggested that the FAC has potentiality to equip cells with proper defense and tolerance ability toward adverse condition. To put forward, ideal host response to commensal microbes in the intestine should be strictly controlled for avoiding inflammation, though it is a highly complex interaction, the main colonized bacteria would be one of the indices to grab the approximate immune condition of the host [18]. In our study, suppressed cecal coliform count and improved ileal and cecal lactic acid bacteria count were found in both 21 and 35 d old birds fed 10% FAC containing diet. Such balanced intestinal microbiota indirectly and partially indicates a relatively integrate immune function [10]. These results could be due to the immune enhancing and antibacterial activities of polysaccharides characterized in previous literature [19]. Non-digestible carbohydrates, such as polysaccharides, are broken down and oxidized incompletely in the lumen by the intestinal microbiota. The subsequent selective exploitation of the fermented product- SCFAs provides a preferable condition for beneficial bacteria, like lactic acid bacteria, yet unfavorable to harmful bacteria [20]. Furthermore, do Nascimento et al [21] corroborate the effective eliminating effects of ursolic acid, a representative pentacyclic triterpenoid, on 6 out of 12 tested bacteria, which included two common strains of *Escherichia coli*. Since a class of triterpenoids produced by AC belong to the same category of triterpenoids widely investigated [22], our results were possibly in line with previous literatures. To support the above results, an important check – growth performance should be considered. Receiving diet with FAC, birds showed higher body weight especially in 10% FAC group than those consumed non-fermented WB. Few research directly indicates the actual adverse action while using WB in chicken diet; nevertheless, hardly digestible NSP WB prone to cause suppressed growth performance in chicken [23]. Nevertheless, the anti-nutritional structure in WB might be destructed after solid-state fermented by AC (Supplementary Figure S1). Accordingly, our results imply promising mitigating effects of FAC on oxidation and inflammation.

In order to achieve equal energy level of diets among dif ferent treatments, larger amount of soybean oil was used in groups containing WB or FAC (Table 2). However, high level of soybean oil, which is rich in polyunsaturated fatty acid (PUFA), is highly susceptible to oxidative deterioration. This may lead to tissue oxidation in animals by negatively affected antioxidant-related genes, such as SOD and CAT [24]. According to our result, the increased SOD and CAT activities suggest that FAC provide a better conquering system preventing birds from tissue oxidation. Interestingly, it’s been reported that high amounts of linoleic acid presented in soybean oils can inhibit effective production of eicosapentaenoic acid, an essential long chain n-3 fatty acids for chicks’ growth, through conversion of α-linolenic acid, which could be attributed to the undesirable modulation of lipogenic enzyme expression that indirectly affect fatty acid composition in animal tissue [25]. In this study, higher levels of monounsaturated fatty acid (MUFA) of the palmitoleic and oleic acid type, and lower levels of saturated fatty acid (stearic acid) was observed in 5% and 10% FAC groups. Moreover, significant reduced omega-6/omega-3 ratio was found in chickens in 5% FAC group. The analysis of fatty acids in breast muscle showed that it is possible to modify the fatty acid profile of the meat, increasing the MUFA content of the oleic acid type and decreasing the saturated fatty acid, by adding FAC in the diet. Additionally, previous literatures indicated that increased proportion of unsaturated fatty acids would promote β-oxidation and uptake of TG in bloodstream [26], and reduced serum cholesterol and LDL. Serum TG level in chickens fed 10% FAC and 5% FAC replaced diet were significantly reduced at 21 and 35 d respectively. Since the clearance of TG and CHO were mainly through HDL carrying to liver for decomposing and expelling [27], the increased high-density lipoprotein cholesterol (HDL-C) implies improvement in CHO clearance and reduction of CHO retention. Moreover, the unsaturated fatty acids, especially n-3 and n-6, are consequential precursors of eicosanoids and prostaglandins that regulate the immunological process [28], and enrichment of PUFA in cell is associated with decreases in the inflammatory response. Our results suggested that AC seemed to provide mechanisms underlying the desirable fatty acid constitution and lipid parameter in the serum, and concomitantly, rendering optimal immune status and improved antioxidant capacity. Other studies applying antioxidants in diets demonstrated that fatty acid profile of meat was affected to a different extent. For instance, Zanini et al [29] found that 400 mg of vitamin E added in diet increased contents of the PUFAs in the chest meat compared to a lower level inclusion of vitamin E in diet (30 mg/kg). Besides, Łukaszewicz et al [30] showed no difference of fatty acid profile of the breast muscle after supplementation of selenium and vitamin to birds’ diet. These, thereafter, proposed that deeper and more upstream ground worth further investigating and elucidating. In conclusion, solid-state fermentation of WB with AC increased the functional metabolites amount in the final product (FAC), possibly related to the optimal intestinal microflora observed in chickens received FAC instead of unfermented WB group. Concomitantly, FAC was equipped with potential antioxidant effects that plausibly exerted in chicken body, indicated by pronounced augmented antioxidant enzymes activities. Moreover, the results further elaborate that FAC would elevate the unsaturated degree of fatty acids in breast muscle, and effectively increased HDL-C level in serum. Finally, improved body weight gain in FAC than WB seemed to accord with those beneficial action, implying promising favorable mechanism endowed by AC.

## Figures and Tables

**Table 1:** Ingredients and chemical composition of the experimental diets for broilers

Items	Starter diet1) (1 to 21 d)					Finisher diet (22 to 35 d)				
	
	Control	5% WB	10% WB	5% FAC	10% FAC	Control	5% WB	10% WB	5% FAC	10% FAC
	---------------------------------------------------g/kg-------------------------------------------									
Corn, yellow	474.4	406.9	339.5	413.1	351.8	520.6	453.2	385.7	459.3	398.1
Soybean meal (CP 44%)	178.5	166.0	153.6	160.7	143.0	103.8	91.3	78.9	86.1	68.3
WB	0	50	100	0	0	0	50	100	0	0
FAC	0	0	0	50	100	0	0	0	50	100
Soybean oil	1.0	29.6	58.3	28.5	56.0	5.7	34.3	62.9	33.2	60.7
Full fat soybean meal	300	300	300	300	300	330	330	330	330	330
Calcium carbonate	16.0	16.0	16.1	16.1	16.1	13.3	13.3	13.3	13.3	13.3
Monocalcium phosphate	18.0	18.5	18.8	18.5	18.8	16.2	16.5	16.7	16.5	16.8
L-lysine-HCl	1.6	2.2	2.8	2.4	3.2	0.9	1.5	2.1	1.6	2.4
DL-methionine	3.7	4.1	4.5	4.2	4.6	3.1	3.5	3.9	3.5	3.9
NaCl	4.0	3.7	3.8	3.7	3.8	3.7	3.7	3.7	3.7	3.7
Choline-Cl	0.8	0.8	0.8	0.8	0.8	0.8	0.8	0.8	0.8	0.8
Vitamin premix2)	1	1	1	1	1	1	1	1	1	1
Mineral premix3)	1	1	1	1	1	1	1	1	1	1
Total	1,000	1,000	1,000	1,000	1,000	1,000	1,000	1,000	1,000	1,000
Calculated nutrient value										
ME (kcal/kg)	3,050	3,050	3,050	3,050	3,050	3,175	3,175	3,175	3,175	3,175
Crude protein (g/kg)	230	230	230	230	230	210	210	210	210	210
Calcium (g/kg)	10.5	10.5	10.5	10.5	10.5	9.0	9.0	9.0	9.0	9.0
Total phosphorus (g/kg)	7.7	7.5	7.4	7.5	7.5	7.1	7.0	7.0	7.0	7.1
Available phosphorus (g/kg)	5.0	5.0	5.0	5.0	5.0	4.5	4.5	4.5	4.5	4.5
Lysine (g/kg)	14.3	14.3	14.3	14.3	14.3	12.5	12.5	12.5	12.5	12.5
Methionine+cysteine (g/kg)	10.7	10.7	10.7	10.7	10.7	9.6	9.6	9.6	9.6	9.6
Analyzed nutrition value										
Crude protein (g/kg)	231	234	232	231	232	213	211	212	212	212
Crude fat (g/kg)	78.6	76.4	76.8	76.6	76.8	85.1	84.6	84.1	84.8	85.3

CP, crude protein; ME, metabolizable energy.

1) WB, wheat bran; FAC, products solid-state fermented wheat bran by *Antrodia cinnamomea* for 16 days.

2) Supplied per kg of diet: Vit A 15,000 IU; Vit D_3_ 3,000 IU; Vit E 30 mg; Vit K_3_ 4 mg; riboflavin 8 mg; pyridoxine 5 mg; Vit B_12_ 25 μg; Ca-pantothenate 19 mg; niacin 50 mg; folic acid 1.5 mg; biotin 60 μg.

3) Supplied per kg of diet: Co (CoCO_3_) 0.255 mg; Cu (CuSO_4_·5H_2_O) 10.8 mg; Fe (FeSO_4_·H_2_O) 90 mg; Zn (ZnO) 68.4 mg; Mn (MnSO_4_·H_2_O) 90 mg; Se (Na_2_SeO_3_) 0.18 mg.

**Table 2:** Crude triterpenoid, total phenolic and crude polysaccharide contents of products solid-state fermented wheat bran by *Antrodia cinnamomea* for 16 days

Items1)	Crude triterpenoid (mg of UAE/g DW)	Crude polysaccharide (mg of GCE/g DW)	Total phenolics (mg of GAE/g DW)
WB	3.48±1.4	60.11±2.9	3.87±0.2
FAC	20.2±1.1	123.3±3.1	16.7±0.7

Values are expressed as the mean±standard deviation of four independent experiment (n = 4).

UAE, ursolic acid equivalent; DW, dry weight; GCE, glucose equivalent; GAE, gallic acid equivalent.

1) WB, wheat bran; FAC, products solid-state fermented wheat bran by *Antrodia cinnamomea* for 16 days.

**Table 3:** Effect of dietary replacement with wheat bran or product solid-state fermented wheat bran by *Antrodia cinnamomea* for 16 days on growth performance of 1–35 d-old broilers

Experimental period (d) and parameter	Experimental diets1)					SEM	p-value

	Control	5% WB	10% WB	5% FAC	10% FAC		
1 to 21 d							
Body weight (g/bird)2)	820^b^	802^b^	763^c^	836^b^	876^a^	12.07	<0.001
Feed consumption (g/bird) 3)	999^b^	907^b^	874^c^	974^b^	1,000^a^	24.09	<0.001
Weight gain (g/bird)2)	775^a^	757^b^	718^b^	791^a^	830^a^	41.85	0.001
FCR3)	1.27^a^	1.26^a^	1.24^ab^	1.23^ab^	1.21^b^	0.031	0.044
22 to 35 d							
Body weight (g/bird)	2,021^a^	1,847^b^	1,823^b^	2,001^a^	2,004^a^	15.51	<0.001
Feed consumption (g/bird)	1,852^a^	1,631^c^	1,632^c^	1,802^ab^	1,774^b^	18.18	<0.001
Weight gain (g/bird)	1,201^a^	1,044^b^	1,061^b^	1,164^a^	1,129^a^	41.41	0.001
FCR	1.51	1.56	1.55	1.52	1.50	0.020	0.392
1 to 35 d							
Feed consumption (g/bird)	2,851^a^	2,499^b^	2,482^b^	2,784^a^	2,777^a^	15.52	<0.001
Weight gain (g/bird)	1,976^a^	1,801^b^	1,766^b^	1,955^a^	1,960^a^	51.60	<0.001
FCR	1.43	1.43	1.41	1.39	1.40	0.034	0.595

SEM, standard error of the mean; FCR, feed conversion rate.

1) WB, wheat bran; FAC, products solid-state fermented wheat bran by *Antrodia cinnamomea* for 16 days.

2) Results are provided as the means of 80 birds in each control and treatment group (n = 80).

3) Results are provided as the means of four replicates (20 birds/replicate) in each control and treatment group (n = 4).

a–c Means within the same rows without the same superscript letter are significantly different (p<0.05).

**Table 4:** Effect of dietary replacement with wheat bran or product solid-state fermented wheat bran by *Antrodia cinnamomea* for 16 days on lactic acid bacteria and coliform count in intestinal content of 21 and 35 days old broilers

Parameter	Experimental diets1)					SEM	p-value

	Control	5% WB	10% WB	5% FAC	10% FAC		
d 21 (log cfu/g)							
Lactic acid bacteria							
Ileum	7.67^c^	9.11^bc^	8.66^c^	9.30^b^	9.35^a^	0.16	<0.001
cecum	9.95^c^	9.60^c^	10.18^b^	11.61^ab^	11.89^a^	0.33	<0.001
Coliform							
Ileum	6.91	7.14	7.24	6.88	6.91	0.32	0.454
cecum	7.85^d^	8.43^a^	8.49^a^	8.26^b^	8.04^c^	0.19	0.012
d 35 (log cfu/g)							
Lactic acid bacteria							
Ileum	6.48	5.88	5.94	6.23	6.31	0.27	0.566
cecum	8.73^a^	8.06^b^	7.98^b^	8.92^a^	8.74^a^	0.15	0.032
Coliform							
Ileum	5.63	6.04	6.13	5.51	5.78	0.17	0.486
cecum	7.96^b^	9.21^a^	9.59^a^	7.82^b^	7.65^b^	0.31	0.012

Results are provided as the means of four replicates (4 birds/replicate) in each control and treatment group (n = 4).

SEM, standard error of the mean.

1) WB, wheat bran; FAC, products solid-state fermented wheat bran by *Antrodia cinnamomea* for 16 days.

a–d Means within the same rows without the same superscript letter are significantly different (p<0.05).

**Table 5:** Effects of dietary replacement with wheat bran or product solid-state fermented wheat bran by *Antrodia cinnamomea* for 16 days on serum lipid parameters (mg/dL) of 21 and 35 days old broilers

Items	Experimental diets1)					SEM	p-value

	Control	5% WB	10% WB	5% FAC	10% FAC		
21 d							
CHO	112.25	119.6	118.23	115.75	108.25	6.37	0.258
TG	62.25^a^	58.03^a^	48.25^b^	49.25^b^	38.10^c^	2.74	<0.001
HDL	81.25^a^	60.33^b^	65.00^b^	81.75^a^	87.75^a^	2.55	<0.001
LDL	21.00^a^	14.67^b^	12.75^b^	11.53^bc^	9.00^c^	1.17	0.014
35 d							
CHO	107.75^ab^	113.75^a^	108.5^ab^	102.25^b^	94.0^c^	3.41	0.006
TG	80.5^a^	53.75^c^	60.25^b^	44.5^d^	52.25^c^	2.61	<0.001
HDL	67.75^b^	68.25^b^	64.0^b^	74.0^a^	76.25^a^	1.60	0.021
LDL	10.75^a^	11.75^a^	6.75^a^	6.25^b^	5.25^b^	1.01	0.016

Results are provided as the means of four replicates (4 birds/replicate) in each control and treatment group (n = 4).

SEM, standard error of the mean; CHO, total cholesterol; TG, triglycerides; HDL, high density lipoprotein; LDL, low density lipoprotein.

1) WB, wheat bran; FAC, products solid-state fermented wheat bran by *Antrodia cinnamomea* for 16 days.

a–d Value within the same row without the same superscript letter are significantly different (p<0.05).

**Table 6:** Effects of dietary replacement with wheat bran or product solid-state fermented wheat bran by *Antrodia cinnamomea* for 16 days (FAC) on fatty acids concentration (%) in pectoral superficial muscle of 35-d-old broiler chickens

Fatty acids	Treatment1)					SEM	p-value

	Control	5% WB	10% WB	5% FAC	10% FAC		
Miristic (C14:0)	0.38	0.4	0.42	0.37	0.38	0.04	0.683
Palmitic (C16:0)	11.32^c^	16.18^b^	18.88^a^	10.69^c^	11.5^c^	0.24	0.004
Palmitoleic (C16:1)	0.11^b^	0.15^b^	0.1^b^	0.87^a^	0.75^a^	0.25	0.013
Heptadecanoic (C17:0)	0.06	0.04	0.05	0.04	0.05	0.01	0.060
Estearic (C18:0)	6.12^b^	6.78^a^	6.68^a^	6.23^b^	6.05^b^	0.2	0.005
Oleic (C18:1n9)	22.93^bc^	23.21^b^	20.68^c^	26.48^a^	26.92^a^	0.25	0.004
Linoleic (C18:2n6)	20.68^ab^	19.22^b^	14.99^c^	23.22^a^	21.69^a^	0.27	0.001
γ-Linolenic (C18:3n6)	0.11^c^	0.14^b^	0.14^b^	0.17^ab^	0.2^a^	0.05	0.043
α-Linolenic (C18:3n3)	2.01^c^	2.12^c^	2.02^c^	3.42^a^	2.68^b^	0.13	0.037
Eicosaenoic (C20:1n9)	0.33	0.36	0.38	0.36	0.34	0.03	0.124
Arachidonic (C20:4n6)	0.25^b^	0.21^b^	0.23^b^	0.38^a^	0.45^a^	0.03	0.004
Docosanoic (C22:0)	0.16	0.21	0.18	0.16	0.18	0.01	0.012
DPA (C11:5n3)	0.03^c^	0.05^bc^	0.05^b^	0.07^a^	0.08^a^	0.02	0.006
DHA (C22:6n3)	0.05^ab^	0.01^c^	0.01^c^	0.07^a^	0.05^b^	0.02	0.010
Saturated	18.04^b^	23.61^a^	26.21^a^	17.49^b^	18.16^b^	0.31	0.015
Monounsaturated	23.37	23.72	21.16	27.71	27.925	0.2	0.124
Polyunsaturated	23.13^b^	21.75^b^	17.44^c^	27.33^a^	24.5^ab^	0.21	0.010
SFA:MUFA:PUFA	1:1.3:1.3	1:1:0.9	1:0.8:0.7	1:1.6:1.6	1:1.5:1.4	-	-
Omega-6	21.04^a^	19.57^b^	15.36^c^	23.77^a^	22.34^a^	0.35	0.043
Omega-3	2.09	2.18	2.08	3.56	2.81	0.28	0.060
Omega-6/Omega-3	10.07	8.98	7.38	6.68	7.95	1.03	0.061

The value is provided as the means of four samples (n = 4).

1) WB, Wheat bran; FAC, products solid-state fermented wheat bran by *Antrodia cinnamomea* for 16 days.

SEM, standard error of the mean; DPA, docosapentaenoic acid; DHA, docosahexaenoic acid; SFA, saturated fatty acid; MUFA, monounsaturated fatty acids; PUFA, polyunsaturated fatty acid.

a–c Value within the same row without the same superscript letter are significantly different (p<0.05).
