# Supplementary material for: Effects of dietary Antrodia cinnamomea fermented product supplementation on antioxidation, anti-inflammation, and lipid metabolism in broiler chickens
Source: Asian-Australas J Anim Sci. 2019 Aug 26;33(7):1113–25. doi: 10.5713/ajas.19.0392 (PMC7322656; doi:10.5713/ajas.19.0392)
Supplement: Supplementary file 1 [file ajas-19-0392-suppl1.pdf]

Suppl. Fig. 1.

(A)

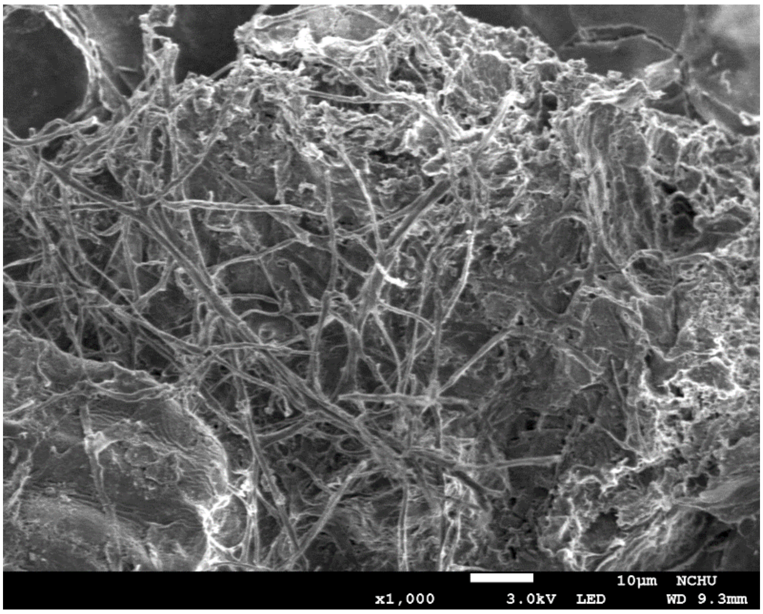

(B)

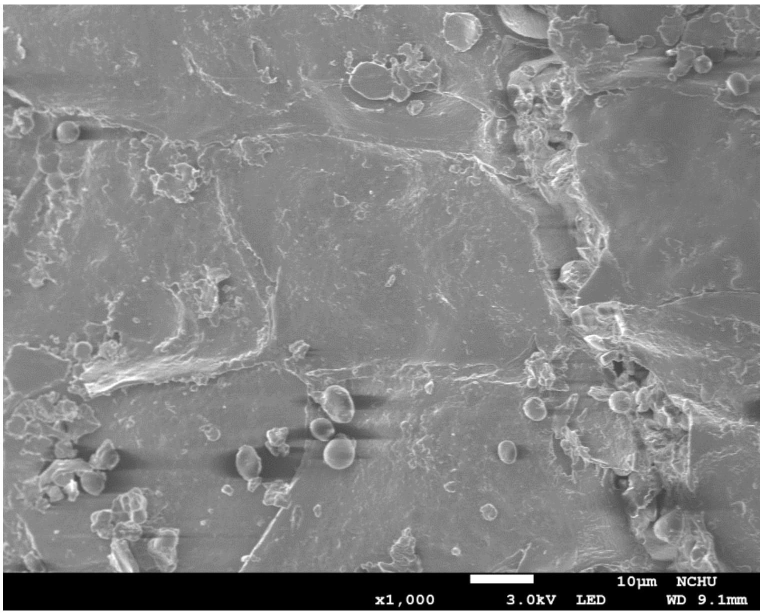

Supplementary Figure S1. Scanning electron microscope micrographs of (A) fermented *A. cinnamomea* from Wang's laboratory (FAC) and (B) non-fermented wheat bran (WB).
